# Describing Inconsistencies in Pediatric Labeling of Medical Devices

**DOI:** 10.1001/jamanetworkopen.2025.46517

**Published:** 2025-12-04

**Authors:** Grzegorz Zapotoczny, Alexis Snitman, Payal Shah, Melissa Bent, Benjamin S. Glicksberg, Florence Bourgeois, Yaniv Bar-Cohen, Juan Espinoza

**Affiliations:** 1 Stanley Manne Children’s Research Institute, Ann & Robert H. Lurie Children’s Hospital of Chicago, Chicago, Illinois; 2 Northwestern University Feinberg School of Medicine, Chicago, Illinois; 3 University of Southern California, Los Angeles; 4 Children’s Hospital Los Angeles, Los Angeles, California; 5 Center for AI in Children’s Health, Icahn School of Medicine at Mount Sinai, New York, New York; 6 Department of Pediatrics, Harvard Medical School, Boston, Massachusetts

## Abstract

**Importance:**

The US Food and Drug Administration (FDA) has recommended, but not required, age ranges for pediatric age labeling in devices, despite regulation existing for drugs, and this policy perpetuates off-label use of devices and creates several issues in practice for safety and monitoring. Proper device labeling is an important regulatory component that impacts risk stratification, clinical use, marketing, and reimbursement.

**Objective:**

To evaluate the variability in pediatric medical device age labeling among all pediatric class III devices approved by the FDA from 2008 to 2017 and their adherence to nonbinding FDA age range recommendations, and to review the device type, clinical use, and whether the pediatric population was included in clinical trials.

**Design, Setting, and Participants:**

This retrospective cross-sectional study reviewed data from public databases to understand pediatric age labeling practices. Data included in the 2008 to 2017 Reports to Congress: Premarket Approval of Pediatric Uses of Devices were reviewed in 2022. Additional data were extracted from the FDA Databases. Two physicians independently reviewed the data and assigned clinical descriptors to each device. Descriptive analysis was performed.

**Exposure:**

Device approval by the FDA from 2008 to 2017.

**Main Outcomes and Measures:**

The primary outcomes were whether approved devices had age labeling, whether they described the approved pediatric population, and whether pediatric populations were included in clinical studies of the devices. Pairwise comparison was done with Student *t* test and *P* < .05 to indicate statistically significant differences.

**Results:**

Of 101 unique devices, 26 (25.7%) did not include any specific age ranges and among the remaining 75 devices, 51 (50.5%) were not indicated for patients younger than 18 years. There was significant variation in age labeling, with only 4 devices using fully structured age ranges (eg, 2 to 17 years). Only 8 (7.9%) devices used the FDA recommended pediatric age ranges. The review of clinical trials evaluating device safety and efficacy revealed that 60 (59.4%) included pediatric patients (aged 0 to <22 years); however, only 33 (32.7%) included patients younger than 18 years. In contrast, the Indications For Use statements for the same devices indicate 52 (51.5%) did not include any specific age ranges and 29 (28.7%) were indicated for adults 18 years or older.

**Conclusions and Relevance:**

In this cross-sectional analysis of devices included the 2008 to 2017 Reports to Congress, most high-risk devices were not evaluated or specifically indicated for children younger than 18 years. This poses substantial problems for clinical use, reimbursement, research, and policy evaluation. To address this gap, the FDA should establish pediatric labeling requirements that consider age and other relevant factors.

## Key Points

**Question:**

What are the current pediatric medical device age labeling practices?

**Findings:**

In this cross-sectional study involving reports from the Food and Drug Administration (FDA) to Congress from 2008 to 2017, 101 unique, high-risk pediatric medical devices were identified, of which only 7.9% used the FDA-recommended age ranges in their Indications For Use statement, whereas 50.5% were labeled for patients aged 18 years and older.

**Meaning:**

These findings indicate that device manufacturers and patients could benefit from FDA-established standardized age-labeling requirements for medical devices, similar to those in drug regulation.

## Teaser

This cross-sectional study examines the variability in pediatric medical device age labeling among all pediatric class III devices approved by the Food and Drug Administration from 2008 to 2017 and their adherence to nonbinding age range recommendations.

## Introduction

Pediatric medical devices play an important role in the diagnosis and treatment of pediatric diseases. Unfortunately, the large majority of medical devices are designed and approved for adults and not for children.^1,2^ This issue can be attributed to a number of complex and interrelated factors, including financial, technical, clinical, and regulatory challenges specific to pediatrics.^2^ Children have distinct clinical, physiological, and developmental needs from adults, leaving pediatric practitioners with few options: (1) use adult devices off-label and either repurpose them or modify them^3^; (2) use older, less effective devices approved for children; or (3) use nondevice alternatives, such as drugs or procedures. A number of legislative initiatives have been introduced over the past 3 decades to address this inequity in pediatric health, but despite some successes, the gap remains.^4^

In the US, medical devices are regulated by the Center for Devices and Radiological Health (CDRH), which is part of the US Food and Drug Administration (FDA).^5^ CDRH is responsible for ensuring the safety, efficacy, and quality of medical devices. A key component of device regulation is labeling. Labeling refers to both the actual label on a device, as well as all of the information about the device that may impact risk stratification, safe and effective clinical use, marketing, and reimbursement.^6^ The general labeling requirements for all medical devices are defined by 21 CFR Part 801 and include requirements for the name and address of the manufacturer, packer, or distributor; description of the intended use and risk classification; directions for use; information on misleading statements; and the use of symbols.^6,7,8^ There are separate requirements for the Unique Device Identifier, over-the-counter devices, exemptions from adequate directions for use, and special requirements for specific devices.^9^

**Figure 1.**
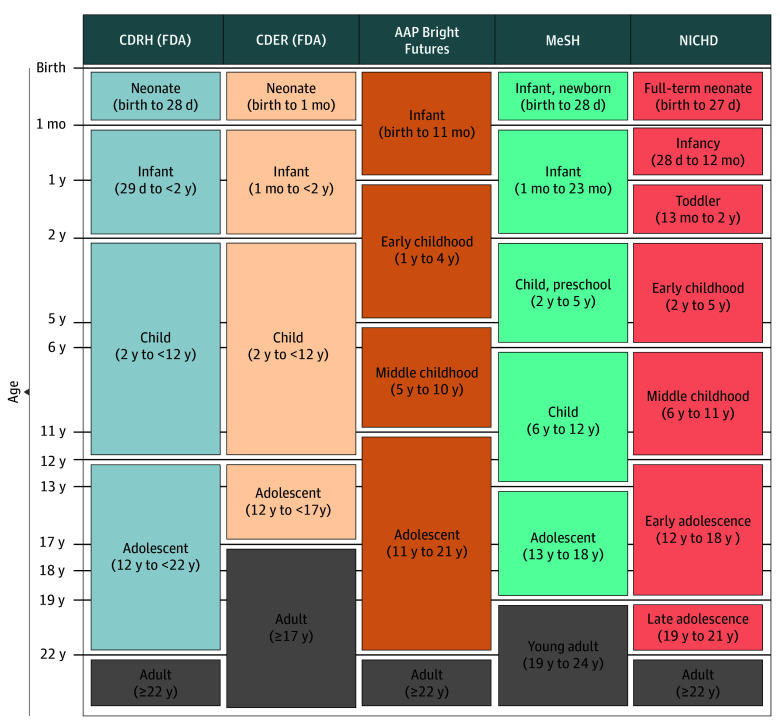
Pediatric Subpopulation Age Ranges by Institution and Authority AAP indicates American Academy of Pediatrics; CDER, Center for Drug Evaluation and Research; CDRH, Center for Devices and Radiological Health; FDA, Food and Drug Administration; MeSH, medical subject heading; NICHD, National Institute of Child Health and Human Development.

As of this writing, there are no requirements to include age or pediatric approval status in the labeling of medical devices. This practice is different from drugs, for which there are pediatric labeling requirements established by the Best Pharmaceuticals for Children Act of 2002, and the Pediatric Research Equity Act of 2003.^10,11^ One critical issue is that there are multiple definitions of pediatrics and pediatric subpopulations, even within the FDA, which defines pediatrics as individuals younger than 22 years, but only for medical devices (Figure 1).^12,13,14,15,16^ Furthermore, because there are no standards or requirements on how to report on age in device labeling, there is substantial variation in how the information is presented when device manufacturers do include it. Finally, there are no structured data or official designation of *pediatrics* in medical devices; thus, it is impossible to accurately track which regulated devices and how many are intended for pediatric use in the US.^17^

The Food and Drug Administration Amendments Act of 2007 requires the FDA to submit annual reports to congress on medical devices approved for pediatrics (0 to <22 years).^18^ These reports focus on the highest-risk devices; those approved via the Premarket Approval (PMA) and Humanitarian Device Exemption (HDE) pathways, which require clinical testing and a comprehensive FDA review for safety. This study aims to (1) describe the types and uses of approved medical devices included in the 2008 to 2017 FDA pediatric Reports to Congress, (2) describe the patterns and variations in age labeling among these devices, and (3) evaluate their adherence to nonbinding FDA age range recommendations.

## Methods

### Data Sources

In 2022, we reviewed all of the publicly available FDA PMA of Pediatric Uses of Devices annual Reports to Congress from 2008 to 2017.^19^ The PMA or HDE number provided in the reports was used to search the FDA PMA^20^ and HDE^21^ databases and analyzed in combination due to small sample sizes. When available, the approval order, summary of safety and effectiveness, pediatric use information and Indications for Use (IFU) statements were used to extract additional information on each device. For devices that reported the ClinicalTrials.gov registration number for the clinical study associated with the device, the information on the pediatric inclusion and involvement was also accessed. The study used publicly available information, did not involve the recruitment of human participants, and as such did not meet the definition of human participants research, and did not require institutional review board approval or informed consent.

### Data Extraction and Validation

Data were extracted from the FDA reports and databases concerning the device regulatory submission and entered into an online database (Airtable). Devices that appeared more than once in the reports due to new supplements being released were included only once in the analysis. J.E. and G.Z. were responsible for performing quality checks on data extraction. Once completed, 2 pediatric physicians (J.E. and M.B.) independently reviewed the data and assigned clinical descriptors to each device.

The clinical trial assessment was conducted by reviewing the information provided by the sponsor to the FDA in the submission materials and the ClinicalTrials.gov entries when the national clinical trial numbers were associated with the device. For the FDA-submitted materials, clinical study details concerning the age ranges of the participants were extracted from the summary of safety and effectiveness. If the pediatric populations as defined by the FDA (0 to <22 years old) were included, we differentiated between inclusion of participants aged 0 to less than 18 years and 18 to less than 22 years. To describe the specificity of age labeling practices, we categorized the data as structured if specific numerical values were used (eg, 4 years old), or unstructured if descriptive, concept-based terms (eg, children) were used. Finally, on the basis of the above information, we noted age labeling variation patterns and defined them as fully structured, partially structured, unstructured, and no specific age ranges as applicable. We defined lower bound as the youngest age the device was approved for and upper bound for the oldest age the device was approved. The ClinicalTrials.gov entries were analyzed and determinations were made according to the available data to assign each entry a categorical variable in regards to the inclusion of pediatric populations, which ClinicalTrials.gov defines as 0 to less than 18 years.

### Statistical Analysis

Descriptive statistical analysis was performed using Excel software (Microsoft). Pairwise comparison was done with Student *t* test, and 2-sided *P* < .05 indicated statistically significant differences.

## Results

### Device Regulatory and Clinical Characteristics

**Figure 2.**
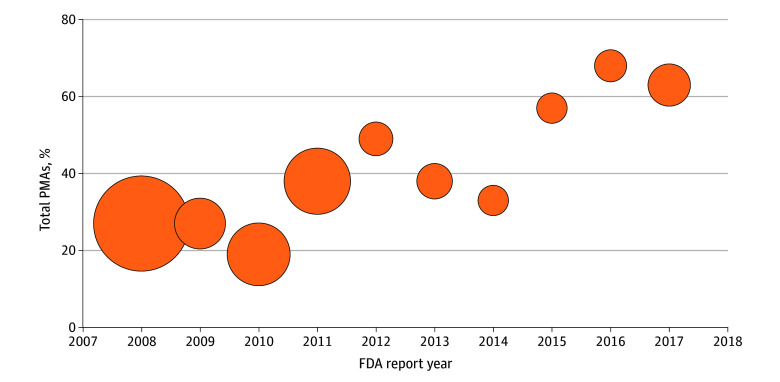
Percentage of Pediatric Devices Approved (Premarket Approvals [PMAs]) Each Year Between 2008 and 2017 Bubble size represents the percentage of all PMAs in a given year that are pediatric. An additional 26 devices did not specify an age range. Of the devices for adolescents, 43 were recommended for patients aged 18 years or older and 8 devices for patients 21 years and older. FDA indicates Food and Drug Administration.

A total of 117 device regulatory submissions approved for use in pediatric patients were identified from reports between the years of 2008 to 2017. Of those, 101 represented unique devices included in our analysis: 90 PMAs and 11 HDEs. The number of total PMAs generally increased from year to year (total, 419 PMAs), but the percentage of those that were approved for pediatric use decreased over the same time period (Figure 2). Among the pediatric PMA devices, 64 (71.1%) were therapeutic, 25 (27.8%) were diagnostic, and 1 (1.1%) was both. Of 11 HDEs, 9 (81.8%) were therapeutic, 1 (9.1%) was diagnostic, and 1 (9.1%) was both.

The Table describes the characteristics of both HDE and PMA devices in detail. On the basis of FDA’s clinical review panel, the most common were cardiovascular devices (24 devices [23.8%]), infectious disease diagnostics (23 devices [22.8%]), and surgical devices (17 devices [16.8%]). In terms of clinical setting, the most prevalent were implantable devices (36 devices [35.6%]) and laboratory tests (27 devices [26.7%]). Twelve devices (11.9%) were reported as combination products (ie, a device with a drug or a biologic component).

**Table.** Characteristics of Pediatric Devices Included in the 2008 to 2017 Reports to Congress

Description	Devices, No. (%) (N = 101)
Regulatory application type	
Premarket Approval	90 (89.1)
Humanitarian Device Exemption	11 (10.9)
Regulatory process metrics	
Food and Drug Administration review time, median (IQR), d	180 (177-249)
Total review time, median (IQR), d	422 (243-697)
User fee exemption granted	5 (5.0)
Expedited review granted	9 (8.9)
Device type	
Diagnostic	26 (25.7)
Therapeutic	73 (72.3)
Both	2 (2.0)
Clinical area	
Cardiovascular	24 (23.8)
Infectious disease diagnostics	23 (22.8)
Endocrinology	11 (10.9)
Surgery	17 (16.8)
Ear, nose, and throat	5 (5.0)
Neurology	5 (5.0)
Ophthalmology	4 (4.0)
Clinical setting or use case	
Implantables	36 (35.6)
Laboratory tests	27 (26.7)
Ambulatory	17 (16.8)
Operating room or procedure room	19 (18.8)
Inpatient critical care	2 (2.0)

The median (IQR) number of days from filling to device approval was 422 (243-697); however, the median (IQR) number of FDA review days for all devices was 180 (177-349). The FDA review was significantly longer for therapeutic devices (248 days) vs diagnostic devices (175 days), but there was no significant difference in the time from filling to approval by device type. Expedited review was granted to 9 devices.

### Age Labeling Patterns in Reports to Congress

**Figure 3.**
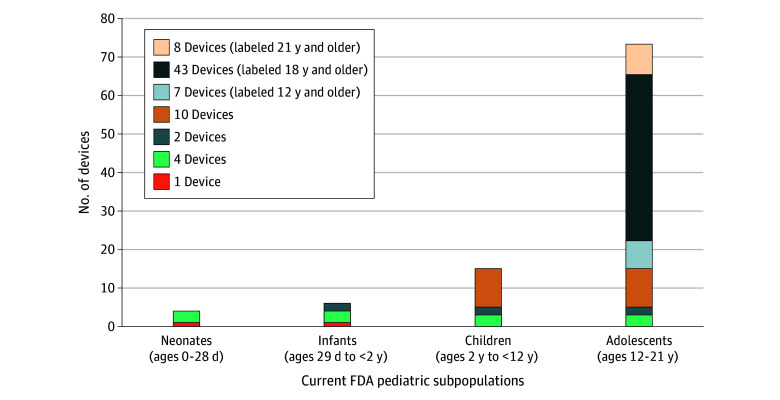
Proportional Area Chart Demonstrating the Intended Patient Populations for the Included Medical Devices, Divided Into the Food and Drug Administration (FDA)–Recommended Age Ranges

The review of the devices’ indications in the reports revealed that of 101 devices, 26 (25.7%) did not include specific age ranges (Figure 3); 10 included pediatric populations generically (eg, all ages, or pediatric and adult patients), and 16 were silent on age. Of the remaining 75 devices, 51 (50.5%) were not indicated for patients younger than 18 years, and 58 devices (77.3%) were approved for use in adolescent patients only (12-21 years old). Of those, 51 (87.9%) were approved for patients 18 and older. Only 5 devices (4.9%) specifically included neonates, and 7 (6.9%) infants. Only 8 devices (7.9%) used the FDA recommended pediatric age ranges. Only 1 device was exclusively pediatric, and 24 were described as being for children younger than 18 years.

Among the 75 devices that specifically included pediatric age labeling, meaning either the lower or the upper bound of the indicated age range implied the device can be used in patients aged 0 to less than 22 years, a structured lower bound was seen in a majority (70 devices [93.3%]), while 8 (10.7%) had a structured upper bound. Consequently, the majority of devices had a partially structured age range with only 4 devices featuring a numerical value at both the upper and lower bounds. Eight devices were found to use the FDA (CDRH) recommended age ranges for medical devices: all of them were indicated for adolescents, 7 included children, and 2 included neonates and infants. The eTable in Supplement 1 describes the patterns and variations in pediatric age labeling.

### Pediatric Inclusion and Labeling Across Regulatory Documents

We identified discrepancies when comparing how pediatric patients were identified and labeled across 3 different sets of regulatory documents: (1) Summaries of Safety and Effectiveness (SSE), (2) IFU Statements, and (3) the Reports to Congress (Figure 3). According to the SSE, only 60 of 101 devices (59.4%) included participants younger than 22 years in their studies, 35 devices (34.7%) did not include any pediatric participants, and the remaining 6 devices (5.9%) did not have age-related data or the median age reported did not allow to draw conclusions on pediatric participation. Only 33 devices (32.7%) included participants younger than 18 years, among which 2 reported a single participant age 17 years, and 1 reported age as a mean (28.7 [18.1] years).

**Figure 4.**
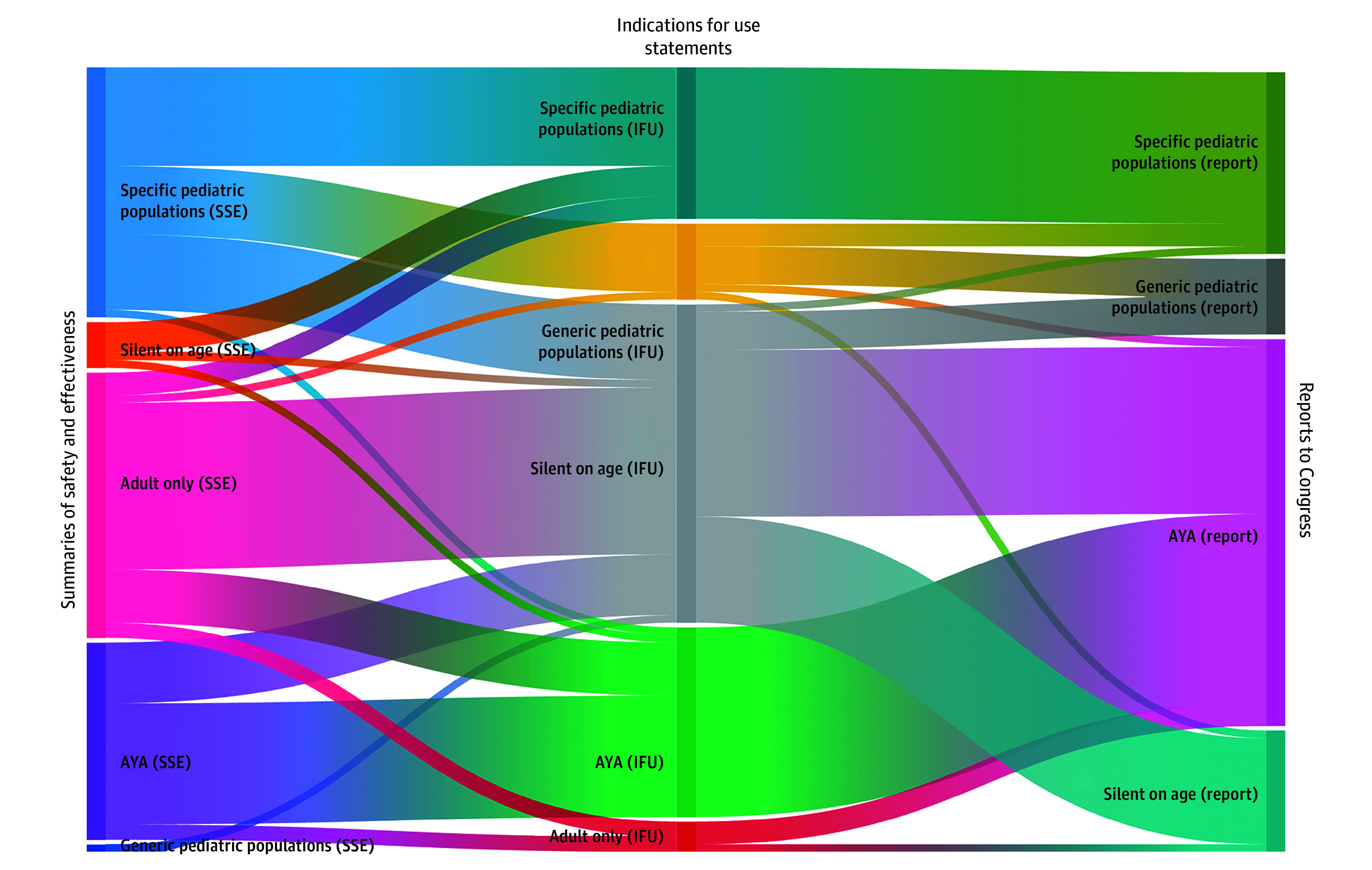
Sankey Diagram Illustrating Variation in Pediatric Labeling Across Regulatory Documents AYA indicates Adolescent & Young Adults Technologies; IFU, Indications for Use; and SSE, Summaries of Safety and Effectiveness.

Despite 33 SSE including pediatric participants younger than 18 years, only 13 (39.4%) were approved with IFUs listing specific pediatric age ranges; the rest either had generic pediatric IFUs, were silent on age, or were approved only for patients aged 18 years and older. Interestingly, 4 SSE that were silent on age and 3 that only included adults were approved with IFUs listing pediatric patients younger than 18 years. Eight SSEs that included adolescents and young adults received IFUs that were silent on age, and 2 had IFUs that were explicitly for adults only. Of the 42 IFUs that were silent on age, only 14 (33.3%) were described in the Reports to Congress as being silent on age, and 22 (52.4%) as suitable for patients 18 years or older, the latter constituting 43.1% of all the Adolescent & Young Adults Technologies (Figure 4).

Pediatric-specific labeling of medical devices included in the Reports to Congress, whenever present, was primarily age based; however, the use of other descriptors to further define the intended patient population was observed and compared in 5 devices. Body surface area (1 device) was used consistently across the IFU statements and the reports. Weight was listed as criteria in the IFU of 3 devices, but that information was not reflected in the Reports to Congress. In the remaining example (HDE number H120005), the report listed weight (21 kg) and age (2 years) requirements, but these could not be found in the IFU, SSE, or any other publicly facing FDA’s documentation.

### Clinical Trial Data

To further investigate the inclusion of pediatric patients in the validation of these medical devices, we reviewed ClinicalTrials.gov entries for the 41 devices that listed the national clinical trial numbers in their regulatory submissions. We identified 38 unique clinical studies (3 clinical trials were associated with more than 1 device each; 1 device listed 2 clinical studies). The ClinicalTrials.gov data are typically presented as the number of participants in each age category: “≤ 18 years,” “between 18 and 65 years,” and “≥ 65 years.” For the purpose of this analysis we considered participants in the 18 years or younger category as pediatric. Of the 38 device studies, 9 (23.0%) had no results posted, and 23 (60.5%) either did not include pediatric patients or their participation was highly unlikely on the basis of the 18 years and older inclusion criteria and the average age of the participants reported. Interestingly, 2 studies reported recruiting a single participant younger than 18 years despite listing 18 years and older as the main age inclusion criterion. Only 4 studies (10.5%) recruited more than 1 pediatric patient with a single study composed exclusively of pediatric patients.

## Discussion

It is estimated that 60% to 75% of medical devices or drugs are used off-label.^22^ The issues stemming from the deficiencies in pediatric drug labeling had been documented 30 years ago and subsequently addressed.^23^ However, unlike in drug labeling regulations (21 CFR Part 201), the 21 CFR Part 801 on device labeling does not mandate the inclusion of age for a given indication.^8^ This poses major problems for clinical use, reimbursement, research, and policy evaluation, as pediatric devices continue to lag behind adults by as much as 10 years.^2,3,24,25,26^

The aim of this cross-sectional study was to evaluate variability in medical device age labeling among PMA and HDE devices reported to Congress by the FDA as pediatric. Through this research, we identified several themes.

First, the current definition of pediatrics for medical devices (0 to <22 years) masks the fact that very few devices are intended for the youngest patients. Of the 101 analyzed devices, only 1 was exclusively pediatric, 7 were reported as suitable for infants, and 24 were described as being for children younger than 18 years. The apparent scarcity of truly pediatric technologies has been well documented by us and other researches documenting pediatric device inequities.^27,28^ Some devices labeled as adult only or for 18 years and older were also included in the reports because of their potential applications in children, such as the gastric bands (to address rising pediatric obesity), breast implants (to treat congenital deformities or posttrauma or burn reconstruction), and intraocular lenses (for congenital or traumatic cataracts).

Second, there is substantial variability in pediatric labeling practices, including instances when age is not mentioned at all. Age labeling guidance for drugs and biologics was finalized in March 2019^10^ and notes that FDA generally recommends using the phrases “pediatric patients X to Y years old,” “pediatric patients aged X to Y years,” or “pediatric patients aged X years and older.” FDA does not currently have similar standards for medical devices, as evidenced by the fact that 51.5% of devices in this study did not contain specific age range information in their IFU statements. The stark contrast between how devices were labeled during the initial FDA approval and how they were later presented to Congress underscores the importance of device labeling and reveals how inadequate the current practices are. The omission of age in noteworthy, as the FDA recognizes that age, among other variables, may affect device performance.^29^

Providing more detailed information in the IFUs would likely require device manufacturers to prioritize children in the design, development, and testing of new devices. The requirements and incentives could be similar to those created for drug manufacturers under the 2002 Best Pharmaceuticals for Children Act and the 2003 Pediatric Research Equity Act.^30^ Our study revealed that pediatric patients aged 0 to 17 years were inconsistently recruited in clinical studies, present in only 32.7% of SSEs. Even by CDRH’s expanded definition of pediatrics, 34.7% devices did not include participants younger than 22 years. Likewise, the ClinicalTrials.gov registration records confirmed that 60.5% of studies did not recruit pediatric patients aged 0 to 17 years. These findings are consistent with previously reported data indicating 84% of high-risk medical device studies did not include any participants younger that 18 years.^24^ The inclusion of children in clinical studies did not always result in a pediatric-specific indication, which further convolutes the issues of what makes a device pediatric.

Finally, it is important to note that physiological and neurodevelopmental changes occur on a spectrum and age is an imprecise proxy for these. For some devices, physiologic, anthropometric, or developmental criteria may be more appropriate markers of inclusion than age. We identified 5 devices in this dataset with mentions of physiologic descriptors to supplement age-based requirements, but their use was inconsistent across sources. Interestingly, the Reports to Congress sometimes either omit these elements or reinterpret them, presumably in an effort to standardize reporting format or to simplify technical information for Congress. Additionally, the relationship between the clinical study data and the selection of physiologic descriptors (or their values) for the IFU statements could not be readily ascertained, such as for a clinical trial of older adults no lighter than 36 kg, precipitated in the IFU for 2 versions of the device, including 1 for patients aged 1 to 8 years, but no heavier than 25 kg.

These themes underscore that clear pediatric labeling guidance for medical devices is paramount to ensure patient safety, especially for high-risk devices. As we attempt to encourage and incentivize pediatric device innovation, it is important to provide clearer and more functional device labeling guidance. From the policy-making standpoint, we recommend that pediatric labeling should be a 2-step process. The first consideration should be whether the device could benefit patients ages 17 years or younger. If affirmed, the next step would be to identify the appropriate inclusion criteria (eg, age, weight, and developmental stage) for pediatric patients that reflects the safety and efficacy data for the device. The current FDA-recommended pediatric subpopulations are neither routinely used in device labeling nor do they map to established developmental milestones. Importantly, all of this information should be captured in structured data formats rather than text or PDFs in order to improve the usability of FDA databases and facilitate reporting and monitoring. Finally, for existing adult devices used in children off-label, real-world data could be leveraged to further investigate device safety and support labeling expansion to include certain pediatric populations.^31,32^

### Limitations

This study has limitations that should be mentioned. The data from the source documents, FDA Reports to Congress and PMA/HDE applications, have been manually extracted as much of the data are in PDF files rather than structured, searchable fields. The data elements in the PMA supplements were difficult to identify and the information was sometimes incomplete. The Reports to Congress used in this study were created manually by CDRH staff. Because the FDA has no internal or public flag or data element that marks a device as being pediatric, it is possible that some relevant devices were not included in the Reports to Congress and, therefore, were not included in this study. Congress directed the FDA to include devices with potential pediatric applications even if not explicitly labeled for pediatric use, which led to the inclusion of devices that are labeled for adults only. Explicit criteria used for such inclusion was not included in the reports. Moreover, the reports evolved in both format and content over time, with earlier years generally providing less detail than later iterations, which limits consistency in longitudinal comparisons. Still, these reports represent the most definitive list of pediatric high-risk devices approved by the FDA since 2008.

Because the Reports to Congress focus on PMA and HDE devices, we did not include class I or II devices in this analysis, despite the fact that they make up almost 90% of all medical devices. Class I devices generally lack formal labeling or IFUs, making it impossible to assess pediatric status from regulatory documentation. For class II devices, there are tens of thousands of products on the market with varying amounts of regulatory information depending on their regulatory pathway, and no structured or standardized labeling regarding pediatric use exists. Conducting a comprehensive review of class II devices would therefore require a large-scale effort well beyond the scope of this study. Future opportunities may lie in applying artificial intelligence and machine learning approaches to FDA databases and publicly available regulatory documents to better identify and classify pediatric devices across all regulatory classes.

## Conclusions

In this cross-sectional analysis of the 2008 to 2017 Reports to Congress on pediatric devices, the results highlighted the need for FDA policy changes and device-specific guidance documents related to age labeling for medical devices. Improvements to FDA data infrastructure could facilitate future research and other quality control initiatives focused on pediatric medical devices. This would provide an opportunity to improve the current labeling practice, establish standard requirements for reporting and labeling, and improve the submission process to create meaningful data that can be easier to navigate in the future. This would also enhance our ability to better understand the availability of pediatric medical devices, and monitor the impact of policy changes over time.

## Notes

Supplement 1. eTable. Patterns of variation in the age labeling of the included pediatric medical devices

Supplement 2. Data Sharing Statement
