# Supplementary material for: Describing Inconsistencies in Pediatric Labeling of Medical Devices
Source: JAMA Netw Open. 2025 Dec 4;8(12):e2546517. doi: 10.1001/jamanetworkopen.2025.46517 (PMC12679324; doi:10.1001/jamanetworkopen.2025.46517)
Supplement: Supplement 1. — eTable. Patterns of variation in the age labeling of the included pediatric medical devices [file jamanetwopen-e2546517-s001.pdf]

Supplemental Online Content

Zapotoczny G, Snitman A, Shah P, et al. Describing inconsistencies in pediatric labeling of medical devices. *JAMA Netw Open*. 2025;8(12):e2546517.
doi:10.1001/jamanetworkopen.2025.46517

eTable. Patterns of variation in the age labeling of the included pediatric medical devices

This supplemental material has been provided by the authors to give readers additional information about their work.

© 2025 Zapotoczny G et al. *JAMA Network Open*.

eTable. Patterns of variation in the age labeling of the included pediatric medical devices.

Lower bound (the youngest the device was approved for) and upper bound (the oldest the device was approved for)

**Characteristics**	**n**	**Examples**
**Lower Bound**		
Structured (discrete number)	70	*"18 months" , "16 years"*
Unstructured (concept)	5	*"Birth", "and younger"*
Not Mentioned	26	*NA*
**Upper Bound**		
Structured (discrete number)	8	*"one year", "17 years"*
Unstructured (concept)	67	*"and older", "greater than"*
Not Mentioned	26	*NA*
**Labeling Variation Patterns**		
Fully Structured Age Range	4	*"2 to 17", "18 months to 21 years"*
Partially Structured Age Range	70	*"18 and younger", "12 and older"*
Unstructured Age Range	1	*"neonatal and older"*
No Specific Age Range	26	*"pediatric patients", "all ages"*

© 2025 Zapotoczny G et al. *JAMA Network Open*.
