# Supplementary material for: Describing Inconsistencies in Pediatric Labeling of Medical Devices
Source: JAMA Netw Open. 2025 Dec 4;8(12):e2546517. doi: 10.1001/jamanetworkopen.2025.46517 (PMC12679324; doi:10.1001/jamanetworkopen.2025.46517)
Supplement: Supplement 2. — Data Sharing Statement [file jamanetwopen-e2546517-s002.pdf]

Data Sharing Statement

Zapotoczny. Describing Inconsistencies in Pediatric Labeling of Medical Devices. *JAMA Netw Open*. Published December 03, 2025. doi:10.1001/jamanetworkopen.2025.46517

Data
Data available: Yes
Data types: Data (not involving human participants)
How to access data: Data available upon request: jespinozasalomon@luriechildrens.org
When available: With publication

Supporting Documents
Document types: None

Additional Information
Who can access the data: anyone requesting the data
Types of analyses: any purpose
Mechanisms of data availability: website link
